# Development of Deltamethrin-Laced Attractive Toxic Sugar Bait to Control *Aedes aegypti* (Linnaeus) Population

**DOI:** 10.1155/2024/6966205

**Published:** 2024-01-06

**Authors:** Sarita Kumar, Aarti Sharma, Roopa Rani Samal, Vaishali Verma, Ravinder Kumar Sagar, Shri Pati Singh, Kamaraju Raghavendra

**Affiliations:** ^1^Department of Zoology, Acharya Narendra Dev College, University of Delhi, Kalkaji, New Delhi 110 019, India; ^2^ICMR-National Institute of Malaria Research, Sector 8, Dwarka, New Delhi 110 077, India

## Abstract

**Background:**

The attractive toxic sugar bait (ATSB) is a promising strategy for controlling mosquitoes at the adult stage. The strategy is based on the use of a combination of fruit juice, sugar, and a toxin in order to attract and kill the adult mosquitoes. The selection of the components and optimization of their concentrations is significant for the formulation of an effective ATSB.

**Methods:**

The present study formulated nine ATSBs and evaluated their efficacy against two laboratory strains (AND-*Aedes aegypti* and AND-*Aedes aegypti-*DL10) and two wildcaught colonized strains of *Aedes aegypti* (GVD-Delhi and SHD-Delhi). Initially, nine attractive sugar baits (ASBs) were prepared using a mixture of 100% fermented guava juice (attractant) with 10% sucrose solution (w/v) in 1 : 1 ratio. ATSBs were formulated by mixing each ASB with different concentrations of deltamethrin in the ratio of 9 : 1 to obtain final deltamethrin concentration of 0.003125–0.8 mg/10 mL ATSB. Cage bioassays were conducted with 50 mosquitoes for 24 h in order to evaluate the efficacy of each ATSB against the four strains of *Ae. aegypti*. The data were statistically analyzed using PASW software 19.0 program and 2-way ANOVA.

**Results:**

The ATSB formulations registered 8.33–97.44% mortality against AND-*Aedes aegypti* and 5.15–96.91% mortality against AND-*Aedes aegypti*-DL10 strains of *Ae. aegypti*, while GVD-Delhi strain registered 2.04–95.83% mortality and SHD-Delhi strain showed 5.10–97.96% mortality. The administration of 0.8 mg of deltamethrin within 10 mL of attractive toxic sugar bait (ATSB) has led to the maximum mortality rate in adult mosquitoes.

**Conclusions:**

The ATSBs formulated with guava juice-ASB and deltamethrin (9 : 1) showed toxin dose-dependent toxicity by all the four strains of *Ae. aegypti.* Most effective dosage was found as 0.8 mg deltamethrin/10 mL ATSB which imparted 96% to 98% mortality in adult mosquitoes. The investigations demonstrated the efficacy of deltamethrin-laced ATSB formulations against *Ae. aegypti* and highlighted the need for conduct of structured field trials and investigating the impact on disease vectors and nontarget organisms.

## 1. Introduction

*Aedes aegypti* (Linnaeus) and *Ae. albopictus* are vectors of global importance for transmission of arboviruses, such as dengue (DENV), chikungunya (CHIKV), Zika (ZIKV), and yellow fever (YFV), while *Ae. vittatus* is reported from few SEAR countries [1]. Lack of effective therapy and vaccination against these arboviruses, except for YFV, has increased the arboviral disease burden worldwide. In addition, emergence and re-emergence of these arboviruses has increased the disease prevalence. Among these, dengue has emerged as one of the fast-spreading diseases with approximately 100–400 million DENV infections/year [2]. The disease is reportedly endemic in more than 100 countries in the 5 WHO Regions with 3.5 billion people at risk of contracting dengue fever and 1.3 billion people living in dengue-endemic areas in 10 countries of South-East Asia Region (SEAR), except in DPR Korea.

In the absence of effective medication and vaccines, *Aedes-*borne diseases are presently managed by vector control [2]. Of the different mechanical, biological, and chemical methods in use, *Aedes* control is still reliant on the chemical-based interventions [3]. Unfortunately, the continued use of these chemicals over a long period of time has caused evolution of insecticide resistance in different mosquito species, including in *Aedes* spp. [4, 5] that retard the disease control.

Use of attractive toxic sugar baits (ATSB) is a relatively new and effective strategy that consists of three components: sugar as a phago-stimulant, a toxin, and an odorant for alluring feeding [3, 6, 7]. The mosquitoes are attracted towards the bait because of the odour of fruit/flower juice and are killed on ingesting the toxin [8]. ATSBs, initially formulated as toxic sugar baits (TSBs) without odorant, were unable to attract mosquitoes due to the absence of an attractant [6]. Thus, fermented fruit/flower juices, with potential to lure mosquitoes by the production of attractive volatiles, were added in the formulations as attractants to formulate ATSBs [9, 10]. Use of ATSBs is contemplated as a reliable control method for both male and female mosquitoes as both quest for sugar sources outdoors. Various fruit juices have been assessed for their attractant potential against mosquitoes, such as guava, banana, mango, orange, tomato, watermelon, and papaya juice against *Anopheles arabiensis* [11], mango and guava juice against *Ae. albopictus* [12], guava juice against *An. gambiae* [13], and guava, mango, muskmelon, orange, papaya, pineapple, plum, sweet lemon, and watermelon juice against *Ae. aegypti* and *An. stephensi* [14, 15].

Initial studies on the sugar-baiting methods against *Ae. aegypti* conducted in the laboratory, using malathion [16] and dinotefuran as toxicants, had effectively reduced the population of *An. gambiae* in Mali, West Africa [17]. The boric acid-containing ATSB has been found effective against *Ae. albopictus*, *Ae. taeniorhynchus*, *Ae. japonicus japonicus*, and *Culex nigripalpus* [3, 18]. Likewise, ATSBs laced with insecticides of different classes, namely, bifenthrin, cyfluthrin, deltamethrin and permethrin (pyrethroids), fipronil (phenylpyrazole), chlorfenapyr (pyrrole), imidacloprid and thiamethoxam (neonicotinoids), and spinosad, ivermectin (macrocyclic lactones), were found effective against *Cx. quinquefasciatus*, *An. quadrimaculatus, Ae. aegypti*, and *Ae. taeniorhynchus* [19, 20]. The formulations containing deltamethrin, fipronil, and imidacloprid were found the most effective followed by other pyrethroids, spinosad, and thiamethoxam, while chlorfenapyr and ivermectin containing TSBs registered least efficacy. Among the pyrethroids, deltamethrin and permethrin were reported highly toxic, bifenthrin moderately toxic while cyfluthrin as the least toxic against mosquitoes [19]. The deltamethrin-containing ATSBs have shown good efficacy against *An. stephensi* in laboratory studies [15].

A comparative assessment of three toxic sugar baits against deltamethrin-resistant *Cx. quinquefasciatus* population showed that the bait containing deltamethrin (0.05%, 0.1%) caused a lower mortality than the bait with boric acid (0.5%, 1.0%) or dinotefuran (0.2%, 0.5%) [21]. Cage bioassays with bait containing guava juice with chlorfenapyr 0.5% v/v, boric acid 2% w/v, and tolfenpyrad 1% v/v registered >90% mortality of pyrethroid-resistant *An. arabiensis* and *Cx. quinquefasciatus* [13]. The ATSB formulated with guava juice-ASB and 0.2–2% boric acid or 0.05–0.5% chlorfenapyr caused 100% mortality in the adults of susceptible (Kisumu) and resistant (M'bé) strains of *An. gambiae* at the maximum tested concentration [22].

ATSB formulations can be applied at the target site in a variety of forms, such as in traps, as toxin-incorporated phago-stimulants in bait stations, as baits at the entrance of storm drain system, or by spraying on plants [6, 9, 10, 17]. In addition, researchers have also demonstrated that use of ATSBs in wild has shown minimal harmful impact on non-target arthropods including the beneficial ones [9, 10, 23, 24]. This novel vector control tool could be used effectively in attracting and killing a large number of mosquitoes, demonstrating its effectiveness in both indoor and outdoor environments. Despite its initial success, it is imperative to standardize the process to ensure its seamless integration and effectiveness at a larger scale.

Current study is a step in this direction with nine ATSB formulations containing cane sugar (10% sucrose solution in water as a phago-stimulant), fermented guava juice (attractant), and graded concentration of deltamethrin (toxin). In this study, guava juice-ASB was selected based on our laboratory studies that showed its superior attractancy than the other eight juices prepared from the locally available fruits [14]. The objective of the study was to assess the laboratory efficacy of nine ATSB formulations containing different concentrations of deltamethrin against two laboratory strains (AND*-Aedes aegypti* and AND*-Aedes aegypti-*DL10) and two wild-caught laboratory colonized strains (GVD-Delhi) and (SHD-Delhi) of *Aedes aegypti* to identify the most effective ATSB formulation.

## 2. Materials and Methods

### 2.1. Rearing and Maintenance of *Aedes aegypti* Mosquitoes in Insectary

The cyclic colonies of *Ae. aegypti* mosquito strains were reared and cultured in the insectary in Insect Pest and Vector Control Laboratory at the Acharya Narendra Dev College, University of Delhi, India, since 2009. The rearing conditions are maintained at regulated temperature (27 ± 2°C), relative humidity (80 ± 10%), and photo-period regime (14L:10D). Adults are reared in cotton cloth fabric cages (45 × 40 × 40 cm) and fed on 10% sucrose solution soaked in a cotton swab kept on the cage roof. For egg maturation, female adults are provided with blood meals from an albino mouse. Laid eggs are collected in an ovicup placed in the cage. The eggs are hatched and cultured in plastic trays half-filled with dechlorinated water and provided a mixture of finely ground dog biscuits and yeast 3 : 2 (w/w) for larval nutrition. The pupae are held in the mosquito cage for emergence to adults.

### 2.2. Strains of *Aedes aegypti* Employed in Study

Two laboratory strains and two wild-caught colonized strains were used for the studies (Table 1).

### 2.3. Laboratory Strains

- Insecticide susceptible strain of *Ae. aegypti* (AND-*Aedes aegypti*): the strain was procured in 2009 from ICGEB (International Centre for Genetic Engineering and Biotechnology), New Delhi, India, and established in the laboratory. Since then, it is being maintained without any insecticide selection pressure.
- Deltamethrin larval-selected strain of *Ae. aegypti* (AND-*Aedes aegypti-*DL10): the early fourth instars of the strain procured from ICGEB in 2009 were subjected to deltamethrin selection pressure at the LC_90_ level for 10 successive generations. The deltamethrin susceptibility status of *F*_10_ was computed, and thereafter, each generation is being selected with the computed LC_90_ value of deltamethrin in order to prevent variations in the deltamethrin susceptibility levels. The adults of the strain, however, were susceptible to deltamethrin as they developed only 1.07-fold deltamethrin resistance.

### 2.4. Wild-Caught Colonized Strains

The wild strains have been maintained in the laboratory since May-June 2021. Since then, approximately 42 generations of these wild strains have been passed.

- Govindpuri strain of *Ae. aegypti* (GVD-Delhi): larvae were collected in June, 2021, from the Govindpuri locality of the Southeast Delhi, India, (28.534°N, 77.265°E) and maintained at Acharya Narendra Dev College, India, without insecticide selection pressure.
- Shahdara strain of *Ae. aegypti* (SHD-Delhi): larvae were collected in May, 2021, from the Shahdara locality of the East Delhi, India, (28.689°N, 77.290°E) and maintained at Acharya Narendra Dev College, India, without insecticide selection pressure.

The adult susceptibility data to deltamethrin of the four *Aedes aegypti* strains are presented in Table 1. The adult strains were completely susceptible to deltamethrin. The LT_50_ values were in the range of 4.43 to 8.78 min and LT_90_ values in the range of 8.85 to 15.99 min (Table 1). The LT_90_ values and resistance ratios in these strains, when compared to susceptible AND-*Aedes aegypti* strain, decreased in the order of *Aedes aegypti* (GVD-Delhi) [1.8] > *Aedes aegypti* (SHD-Delhi) [1.68] > AND-*Aedes aegypti-*DL10 [1.12] > AND-*Aedes aegypti* [1.0] (Table 1).

### 2.5. Formulation of Attractive Toxic Sugar Baits (ATSBs)

The guava juice-ASB was prepared by mixing fermented pure juice of guava and 10% sucrose solution (w/v) in 1 : 1 ratio [14]. Deltamethrin was serially diluted in ethanol to obtain the concentrations in range of 0.03125 mg/mL, 0.0625 mg/mL, 0.125 mg/mL, 0.25 mg/mL, 0.5 mg/mL, 1.0 mg/mL, 2.0 mg/mL, 4.0 mg/mL, and 8.0 mg/mL. The ATSB solutions were prepared by mixing 9 mL of guava juice-ASB with 1 mL of deltamethrin solution at a particular concentration (9 : 1 ratio), resulting in the nine ATSB formulations containing deltamethrin in the range of 0.003125–0.8 mg/10 mL ATSB.

### 2.6. Cage Bioassays with ATSBs

The bioassay with each of the nine ATSB formulations was conducted in individual cloth cages (45 × 40 × 40 cm). A total of eighteen (nine controls and nine experimental) cotton discs (5 cm diameter, 1.5 cm thickness, 0.5 g) were prepared. The cotton discs were soaked with 5 mL of 10% sucrose solution in water (w/v), and the experimental cotton discs were soaked with 5 mL of nine (9) different deltamethrin concentration ATSB formulations.

**Figure 1:**
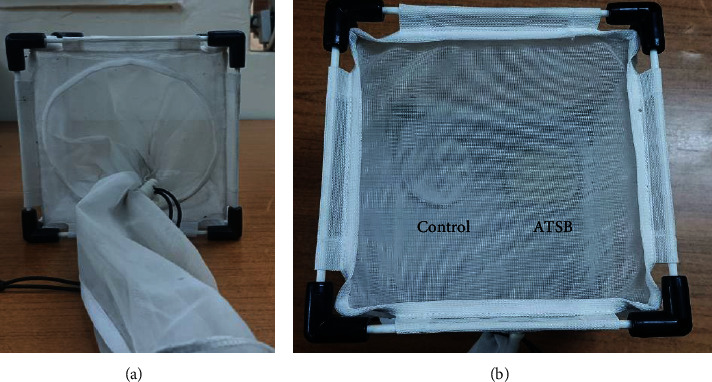
Cage bioassay with *Aedes aegypti* adults: (a) screening cage setup with 50 unfed adult mosquitoes (25 males and 25 females) and (b) screening cage setup with ATSB (guava juice-ASB + deltamethrin) and control (10% sucrose solution) bait placed at two sides.

The assay was conducted in cloth cages of the dimensions (45 × 40 × 40 cm). Earlier studies have shown the high attractant and non-toxic properties of guava juice-ASB [14], and the studies with ATSBs have shown their toxic effects against mosquitoes feeding upon them (unpublished data). To assess the efficacy of an ATSB in a controlled environment, one control disc and one experimental disc of given deltamethrin-ATSB concentration were placed on the two sides of a cage (Figure 1(a)). In each cage, 2-3 days old and unfed fifty *Ae. aegypti* adults, 25 females and 25 males, were released for 24 h (Figure 1(b)). After 24 h exposure, a number of mosquitoes, landed on ATSB and either knocked down (unable to fly but alive) or dead, were recorded and analysed. Four (4) replicates were tested for each concentration of ATSB. Thus, for each strain, a total of thirty-six cages were set-up for ATSB bioassays (9 doses × 4 replicates). Concurrently, control assays were held with attractive sugar baits (ASB) containing fermented pure juice of guava and 10% sucrose solution (w/v) in 1 : 1 ratio.

### 2.7. Statistical Analysis

The percent mortality in adults was calculated in each bioassay by using the following formula (equation (1)):

(1) $$\begin{array}{c} \text{percent mortality }\left(\%\right)=\frac{\text{total number of dead adults }}{\text{total number of exposed adults}}\times 100. \end{array}$$

The control bioassays resulting in >20% mortality were discarded, and experiments with control mortality in the range of 5–20% were corrected using Abbott's formula given in the following equation (2) [25]:

(2) $$\begin{array}{c} p\text{ercent test mortality }\left(\%\right)=\frac{T-C\times 100}{100\;-\;C}, \end{array}$$

where T is the percent mortality of *Ae. aegypti* on the guava juice-deltamethrin-ATSB and C is the percent control mortality of *Ae. aegypti*.

The mortality data were analyzed and interpreted using one-way ANOVA and Tukey's all pairwise multiple comparison test using Predictive Analytics Soft Ware (PASW) 19.0 program.

## 3. Results

The cage bioassays were carried out with ATSB prepared with guava juice-ASB and deltamethrin insecticide (9 : 1) using nine doses of deltamethrin in the range of 0.003125–0.8 mg/10 mL ATSB. Each strain showed a dose-dependent mortality response to deltamethrin-ATSBs with respect to the deltamethrin dosage in the ATSB. After 24 hours of assay, the % mortality in AND-*Aedes aegypti* strain on the ATSBs ranged from 8.33% to 97.44%, while the % mortality in AND-*Aedes aegypti*-DL10 adults was in the range of 5.15% to 96.91% (Table 2). No mortality was recorded in the mosquitoes fed on ASB.

The guava juice-ATSB with the lowest dose of deltamethrin (0.003125 mg/10 mL ATSB) resulted in 8.33% and 5.15% adult mortality in the AND-*Aedes aegypti* and AND-*Aedes aegypti*-DL10 strains, respectively (Table 2). After 24 h of feeding on 0.0625 mg/10 mL and 0.0125 mg/10 mL deltamethrin-ATSB, the observed mortality in AND-*Aedes aegypti* strain was 14.74% and 19.79%, respectively (*P* < 0.05). Likewise, the ATSB formulations containing higher doses of deltamethrin, 0.025 mg/10 mL, 0.05 mg/10 mL, and 0.1 mg/10 mL ATSB, enhanced the adult mortality to 26.53%, 38.38%, and 49.48% in AND-*Aedes aegypti* adults. Similarly, 24 h provision of 0.2, 0.4, and 0.8 mg deltamethrin/10 mL ATSB increased mortality further by 1.35, 1.19, and 1.21-fold in AND-*Aedes aegypti* adults (Table 2). The results showed >80% mortality caused by 0.4 mg deltamethrin-ATSB against AND*-Aedes aegypti* strain.

**Figure 2:**
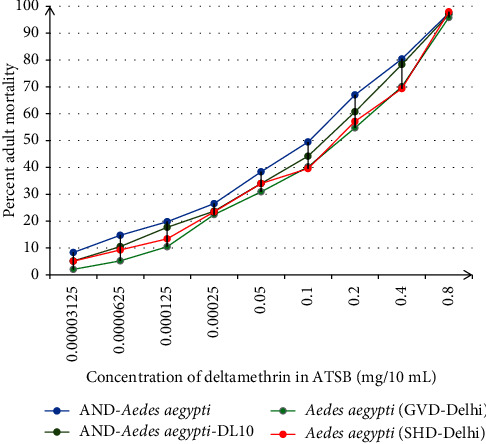
Percent mortality in the adults of laboratory strains (AND-*Aedes aegypti* and AND-*Aedes aegypti-*DL10) and wild-caught laboratory colonized strains (*Aedes aegypti* (GVD-Delhi) and *Aedes aegypti* (SHD-Delhi)) of *Aedes aegypti* exposed to guava juice-deltamethrin-ATSB for 24 h in cage bioassay.

In comparison, relatively lower mortality was recorded in the adults of deltamethrin larval-selected AND-*Aedes aegypti*-DL10 strain (Table 2). Using 0.0625 mg/10 mL and 0.0125 mg/10 mL deltamethrin-ATSB as baits caused 10.53% and 17.71% adult deaths (*P* < 0.05) which enhanced to 23.71%, 34.02%, and 44.21% on providing 0.025, 0.05 and 0.1 mg deltamethrin/10 mL ATSB baits (*P* < 0.05), respectively (Table 2). Similar trend was noticed with 0.2, 0.4, and 0.8 mg/10 mL deltamethrin-ATSB resulting in further increased mortality (1.37, 1.29, and 1.23-fold) in AND-*Aedes aegypti*-DL10 strain, respectively (Table 2, Figure 2), with >80% mortality obtained with 0.8 mg deltamethrin/10 mL ATSB.

The attract and kill potential of nine ATSBs containing 0.003125 to 0.8 mg deltamethrin/10 mL ATSB was also investigated against two wild-caught strains of *Ae. aegypti,* the *Aedes aegypti* (GVD-Delhi), and *Aedes aegypti* (SHD-Delhi) strains which were colonized in the laboratory (Table 3). During 24 h exposure, the ATSB formulations induced 2.04% to 95.83% mortality in GVD-Delhi strain, while comparatively higher mortality of 5.10% to 97.96% was observed in the SHD-Delhi strain of *Ae. aegypti*. The lowest adult mortality rates of 2.04% and 5.10% in *Aedes aegypti* (GVD-Delhi) and *Aedes aegypti* (SHD-Delhi), respectively, were observed with ATSB containing the lowest dose of deltamethrin (0.003125 mg/10 mL ATSB). The increase in the concentrations of deltamethrin in the ATSB formulations increased the adult mortality in both the strains indicating a dose-mortality response correlation.

The 24 h ATSB exposure with 0.0625 mg and 0.0125 mg deltamethrin/10 mL ATSB resulted in 5.21% and 10.47% (*P* < 0.05) adult mortality in GVD-Delhi strain, whereas relatively higher mortality of 9.28% and 13.40% (*P* < 0.05) was obtained in SHD-Delhi strain (Table 3). When exposed to the ATSB formulations with higher doses of deltamethrin (0.025, 0.05, and 0.1 mg/10 mL ATSB), the mortality in *Aedes aegypti* (GVD-Delhi) and *Aedes aegypti* (SHD-Delhi) increased to 22.45–40.21% (*P* < 0.05) and 23.47–39.58% (*P* > 0.05), respectively (Table 3). Mortality increased further in the adults of GVD-Delhi and SHD-Delhi strains by 1.28 and 1.21-folds when exposed to the 0.4 mg deltamethrin/10 mL ATSB with respect to the 0.2 mg deltamethrin/10 mL ATSB; and by 1.36 and 1.41-folds with 0.8 mg deltamethrin/10 mL ATSB in comparison to 0.4 mg deltamethrin/10 mL ATSB, respectively (Table 3, Figure 2). Both the strains registered >80% mortality with 0.8 mg deltamethrin/10 mL ATSB.

**Figure 3:**
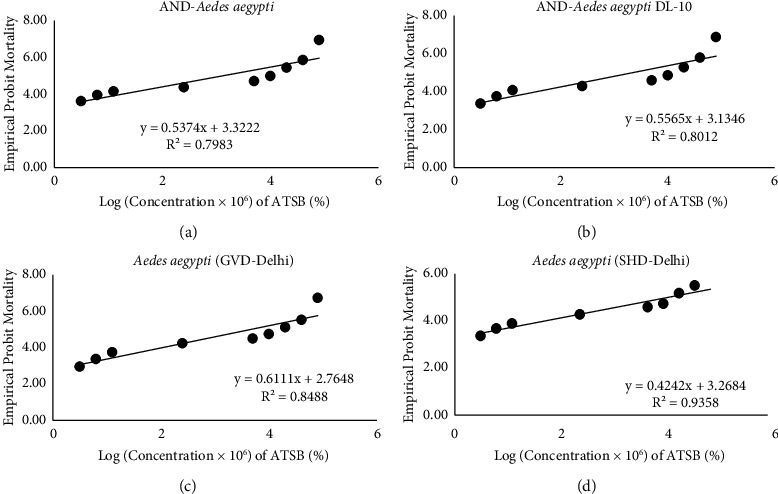
Dosage-mortality regression lines on providing deltamethrin-ATSBs to the laboratory strains (AND*-Aedes aegypti* and AND-*Aedes aegypti*-DL10) and wild-caught colonized strains (*Aedes aegypti* (GVD-Delhi) and *Aedes aegypti* (SHD-Delhi)) of *Aedes aegypti*.

The dose mortality response lines obtained on providing nine deltamethrin-ATSBs to AND-*Aedes aegypti* and AND-*Aedes aegypti*-DL10 showed *R*^2^ values of 0.7983 and 0.8012, respectively (Figures 3(a) and 3(b)) while *R*^2^ values obtained with *Aedes aegypti* (GVD-Delhi) and *Aedes aegypti* (SHD-Delhi) strains were 0.8488 and 0.9358 (Figures 3(c) and 3(d)).

## 4. Discussion

The ATSB is a mixture of three components; fruit juice, a toxin, and sugar solution; to attract for feeding and kill the adults by toxin feed. It is based on the fact that mosquitoes require a sugar diet throughout their life for energy, growth, development, mating, and egg production [8, 26]. Since mosquitoes search for sugar sources in the environment, the ATSB with table sugar competes with the available sources of plant sugar and provides nourishment for survival [6].

The first toxic sugar bait (TSB) was developed against *Ae. aegypti* using malathion and 20% sucrose solution combination [16]. Malathion was added to sucrose in different concentrations (1 mg/mL, 0.5 mg/mL, 0.25 mg/mL, and 0.1 mg/mL) resulting in up to 85.2% adult mortality. However, TSBs, though effective in laboratory evaluation, could not register comparable mortalities in the field due to the presence of the competing environment's natural sugar sources and attractants. Consequently, addition of odour attractants, in the form of fruit juices, flower nectar, or bug honeydew, resulted in the formulation of ATSBs [9, 10, 27]. The laboratory or field trials with different ATSBs have showed varied efficacy which may be not only because of the toxin used but also due to the attractant used, type and prevalence of mosquito species, level of resistance in the mosquitoes to the toxin, and ecological factors. Thereafter, several toxins have been used in the ATSB formulations such as deltamethrin, boric acid, dinotefuran and spinosad [6, 14, 15, 17, 21], fipronil [22, 28, 29], chlorfenapyr and tolfenpyrad [13], eugenol [30], ivermectin [11], sodium ascorbate [31], and microencapsulated garlic oil in beta-cyclodextrin [7, 32–34].

Earlier studies with nine ASBs prepared by combining nine different fermented pure fruits juices with 10% sucrose solution in water in 1 : 1 ratio revealed guava juice-ASB as the most effective attractant for *Ae. aegypti* [14]. The present study is to validate these laboratory results on wild-caught laboratory colonized *Ae. aegypti* strains with nine ATSBs formulated by adding 9 parts of guava juice-ASB to 1 part of various dosages of a contact pyrethroid insecticide, deltamethrin, in the range of 0.003125 to 0.8 mg/10 mL ATSB. These ATSBs were evaluated for their toxic potential against the two laboratory strains (AND*-Aedes aegypti* and AND*-Aedes aegypti-*DL10) and two wild-caught laboratory colonized strains (*Aedes aegypti* (GVD-Delhi) and *Aedes aegypti* (SHD-Delhi)) of *Ae. aegypti.* The studies revealed a dose-dependent mortality response in adult *Ae. aegypti* of ATSBs after 24 h exposure. The recorded mortality in AND-*Aedes aegypti* and AND-*Aedes aegypti*-DL10 ranged from 8.33 to 97.44% and 5.15–96.91%, respectively, whereas these formulations induced relatively less mortality in the laboratory colonized wild-caught deltamethrin susceptible GVD-Delhi strain (2.04–95.83%) and SHD-Delhi strain (5.10–97.96%). The formulations with 0.4 mg deltamethrin/10 mL ATSB caused >80% mortality in the adults of AND-*Aedes aegypti,* while rest of the three strains registered >80% mortality in the adults with 0.8 mg deltamethrin/10 mL ATSB.

The observed mortality in cage bioassays was found to be correlated with the LT_50_ values of deltamethrin obtained against these strains. Increased adult mortality was observed in the AND-*Aedes aegypti* strain (LT_50_ = 4.431 min) followed by AND-*Aedes aegypti*-DL10 (LT_50_ = 4.766 min) and *Aedes aegypti* (SHD-Delhi) (LT_50_ = 8.382 min), while *Aedes aegypti* (GVD-Delhi) (LT_50_ = 8.787 min) showed lowest mortality relative to the other strains. These results are encouraging and need to be validated with the field studies as to date, most of the research in the field of ATSB has been carried out with oral toxins: dinotefuran, spinosad, chlorfenapyr, and boric acid. Contact insecticides-TSBs, though investigated, have been in limited focus.

A few studies have assessed ATSBs formulated using different pyrethroids such as deltamethrin, permethrin, cyfluthrin, and bifenthrin and found them effective against different species of mosquitoes, *Cx. quinquefasciatus, An. quadrimaculatus, An. stephensi, Ae. aegypti,* and *Ae. taeniorhynchus* [15, 19–21]. It was reported that formulations were generally more effective against pyrethroid-susceptible populations than the pyrethroid-resistant populations [21]. Thus, ATSBs containing insecticides with modes of action different from that of pyrethroids were formulated and found effective against pyrethroid-resistant mosquitoes [13]. It has been thus recommended that use of insecticides with an alternate mode of action to the existing insecticide in use should be preferred as a strategy for effective vector management [1].

Current laboratory investigations revealed the efficacy of deltamethrin as a toxin component in the ATSB to control *Ae. aegypti* population. The dosage of 0.8 mg deltamethrin in 10 mL ATSB was found to be highly effective resulting in 96% to 98% mortality in adult mosquitoes. However, reports have suggested the reduced efficacy of ATSBs in the fields in comparison to the laboratory assays probably due to the development of resistant strains [13, 35]. Moreover, it can be due to the availability of natural sugar sources in the natural environment which compete with bait stations.

Based on the encouraging results from this study, it is pertinent to assess the efficacy of the developed deltamethrin-ATSB formulation for use in the field against wild mosquitoes. Further, supplementary studies are recommended on the impact of ATSBs on the environment and non-target organisms, which would help in ascertaining the safe use of ATSBs.

## 5. Conclusions

The study was conducted using nine ATSB formulations with fermented guava juice (100%), sucrose solution (10% w/v), and nine doses of pyrethroid deltamethrin (0.003125–0.8 mg/10 mL ATSB) to assess their toxic potential against two laboratory strains and two wild-caught colonized strains of *Ae. aegypti.* The studies revealed a deltamethrin dose-dependent impact of ATSBs on the mortality in adult mosquitoes. The recorded mortality in laboratory strains, AND-*Aedes aegypti* and adults derived from AND-*Aedes aegypti-*DL10 strain selected with deltamethrin at the larval stage for 10 generations, ranged from 8.33–97.44% to 5.15–96.91%, respectively, whereas these formulations induced 2.04–95.83% and 5.10–97.96% mortality in laboratory colonized wild-caught GVD-Delhi and SHD-Delhi strains of *Ae. aegypti*, respectively. The investigations indicating a positive correlation between the % mortality in the adults and the deltamethrin susceptibility demonstrated the efficacy of these ATSB formulations against *Ae. aegypti* with deltamethrin. This study highlighted the need to conduct structured field trials and investigation of the impact on non-target organisms.

## Figures and Tables

**Table 1:** Deltamethrin susceptibility status of the four strains of *Aedes aegypti*: laboratory strains (AND-*Aedes aegypti* and AND-*Aedes aegypti*-DL10) and wild-caught colonized strains (GVD-Delhi and SHD-Delhi) of *Aedes aegypti* after 24 h exposure to 0.05% deltamethrin-impregnated papers.

Strains	LT_50_ (min.)	RR LT_50_ (min.)	LT_90_ (min.)	RR LT_90_ (min.)	% Mortality after 24 h
AND-*Aedes aegypti*	4.43	—	8.85	—	100
AND-*Aedes aegypti-*DL10	4.76	1.07 (S)	9.98	1.12 (S)	100
*Aedes aegypti* (GVD-Delhi)	8.78	1.98 (S)	15.99	1.80 (S)	100
*Aedes aegypti* (SHD-Delhi)	8.38	1.89 (S)	14.91	1.68 (S)	100

LT_50 or 90_: lethal time for killing 50% or 90% mosquitoes exposed; RR: resistance ratio; S: susceptible, based on the WHO protocol (WHO, 2022).

**Table 2:** Number of adults of laboratory strain (AND*-Aedes aegypti* and AND*-Aedes aegypti-*DL10) of *Aedes aegypti* attracted and killed in ATSB cage bioassays.

ATSB (guava juice-ASB + mg deltamethrin/10 mL)	No. of dead adults ± SE (mortality)	
	AND*-Aedes aegypti*^*∗*^	AND*-Aedes aegypti-*DL10^*∗*^
Control (ASB)	0	0
0.003125	4.16 ± 1.00^a^ (8.33%)	2.57 ± 1.50^a^ (5.15%)
0.00625	7.36 ± 1.00^b^ (14.74%)	5.26 ± 1.00^a^ (10.53%)
0.0125	9.89 ± 1.50^b^ (19.79%)	8.85 ± 0.50^b^ (17.71%)
0.025	13.26 ± 1.00^c^ (26.53%)	11.85 ± 0.50^c^ (23.71%)
0.05	19.19 ± 1.00^d^ (38.38%)	17.01 ± 0.50^d^ (34.02%)
0.1	24.74 ± 2.00^e^ (49.48%)	22.10 ± 3.00^e^ (44.21%)
0.2	33.50 ± 1.50^f^ (67.02%)	30.36 ± 1.50^f^ (60.73%)
0.4	40.20 ± 2.00^g^ (80.41%)	39.17 ± 1.00^g^ (78.35%)
0.8	48.71 ± 0.00^h^ (97.44%)^*∗∗*^	48.45 ± 0.00^h^ (96.91%)

^*∗*^Four replicates each with *n* = 50, 25 males and 25 females (24 h), total *n* = 200. ^*∗∗*^Corrected percent mortality; values in the table represent the number of mosquitoes dead; ATSBs with different letters (column-wise) are significantly different (*P* < 0.05) computed by one-way ANOVA followed by Tukey's all pair wise multiple comparison test.

**Table 3:** Number of adults of wild-caught colonized population of *Aedes aegypti* (GVD-Delhi) and *Aedes aegypti* (SHD-Delhi) strains of *Aedes aegypti* attracted and killed towards ATSB formulation during ATSB cage bioassays.

ATSB (guava juice-ASB + mg deltamethrin/10 mL)	No. of dead adults ± SE (mortality)	
	*Aedes aegypti* (GVD-Delhi)^*∗*^	*Aedes aegypti* (SHD-Delhi)^*∗*^
Control (ASB)	0	0
0.003125	1.02 ± 0.00^a^ (2.04%)	2.55 ± 0.50^a^ (5.10%)
0.00625	2.60 ± 0.50^a^ (5.21%)	4.63 ± 0.50^b^ (9.28%)
0.0125	5.23 ± 0.50^b^ (10.47%)	6.70 ± 1.50^b^ (13.40%)
0.025	11.22 ± 1.00^c^ (22.45%)	11.73 ± 1.50^c^ (23.47%)
0.05	15.46 ± 1.00^d^ (30.93%)	17.01 ± 0.50^d^ (34.02%)
0.1	20.10 ± 1.50^e^ (40.21%)	19.79 ± 1.00^d^ (39.58%)
0.2	27.36 ± 1.00^f^ (54.74%)	28.57 ± 1.00^e^ (57.14%)
0.4	35.05 ± 1.00^g^ (70.10%)	34.69 ± 1.00^f^ (69.39%)
0.8	47.91 ± 0.00^h^ (95.83%)^*∗∗*^	48.97 ± 0.00^g^ (97.96%)

^*∗*^Four replicates each with *n* = 50, 25 males and 25 females (24 h), total *n* = 200. ^*∗∗*^Corrected percent mortality; values in the table represent the number of mosquitoes dead; ATSBs with different letters (column-wise) are significantly different (*P* < 0.05) computed by one-way ANOVA followed by Tukey's all pair wise multiple comparison test.

## Data Availability

All data generated or analyzed during this study are included in the article.

## Abbreviations

AND: Acharya Narendra Dev
ASB: Attractive Sugar Bait
ATSB: Attractive Toxic Sugar Bait
GVD: Govindpuri
SHD: Shahdara
LC: Lethal Concentration
NIMR: National Institute of Malaria Research
TSBs: Toxic Sugar Baits
WHO: World Health Organization
